# A Unidirectional Cell Switching Gate by Engineering Grating Length and Bending Angle

**DOI:** 10.1371/journal.pone.0147801

**Published:** 2016-01-28

**Authors:** Shu Fan Zhou, Singaram Gopalakrishnan, Yuan Hao Xu, Jie Yang, Yun Wah Lam, Stella W. Pang

**Affiliations:** 1 Department of Electronic Engineering, City University of Hong Kong, Kowloon, Hong Kong; 2 Centre for Biosystems, Neuroscience, and Nanotechnology, City University of Hong Kong, Kowloon, Hong Kong; 3 Department of Biology and Chemistry, City University of Hong Kong, Kowloon, Hong Kong; University of Illinois at Chicago, UNITED STATES

## Abstract

On a microgrooved substrate, cells migrate along the pattern, and at random positions, reverse their directions. Here, we demonstrate that these reversals can be controlled by introducing discontinuities to the pattern. On “V-shaped grating patterns”, mouse osteogenic progenitor MC3T3-E1 cells reversed predominately at the bends and the ends. The patterns were engineered in a way that the combined effects of angle- and length-dependence could be examined in addition to their individual effects. Results show that when the bend was placed closer to one end, migration behaviour of cells depends on their direction of approach. At an obtuse bend (135°), more cells reversed when approaching from the long segment than from the short segment. But at an acute bend (45°), this relationship was reversed. Based on this anisotropic behaviour, the designed patterns effectively allowed cells to move in one direction but blocked migrations in the opposing direction. This study demonstrates that by the strategic placement of bends and ends on grating patterns, we can engineer effective unidirectional switching gates that can control the movement of adherent cells. The knowledge developed in this study could be utilised in future cell sorting or filtering platforms without the need for chemotaxis or microfluidic control.

## Introduction

The migration of cells is crucially important to almost all biological functions, including inflammation, wound healing, and metastasis [1–5]. Cell migration is mediated and regulated through the interactions between cell surface receptors and the extracellular microenvironment [5, 6]. These interactions are then transduced intracellularly, via a family of GTPases, to drive the cytoskeletal reorganisation that eventually leads to the net locomotion of cells [7–9]. The intracellular events during cell migration have been delineated in great molecular details, mainly through cell culture studies. Relatively less is known about the roles of the extracellular microenvironment in this process. Concentrations of extracellular biomolecules can be programmed by utilising microfluidic devices, allowing a quantitative study of cell chemotaxis [10–12]. Apart from chemical cues, the mechanical properties of the underlying substrate have also been shown to influence cell migration. For instance, fibroblasts preferred to migrate from soft to stiff regions [13–16]. In addition, migration characteristics under fluid induced shear stress is of particular interest as endothelial cells located in the vessel walls continuously experience the blood flux [17, 18]. Electric field can also direct cell locomotion: both cell motility and directionality can be influenced, in a cell dependent manner, by electric fields with physiological strengths [19–23].

Besides the above-mentioned factors, the topographical features patterned on the underlying substrates can also effectively control cell migration [24–32], among other biological processes [33–39]. While the topography could be fabricated by electrospinning to provide 3D guidance [40, 41], in 2D form, it can be achieved by conventional lithography with precise feature size control. One of the most widely used topography patterns is grating, which is composed of louver-like arrays of ridges. These patterns restrict the lateral protrusion of lamellipodia, thereby favouring the spreading of the cells along the main axis [42–45]. As a result, mammalian cells of various types were observed to elongate and migrate along the grating [46–51]. Our group has been interested in a particular phenomenon of this guided migration: when fibroblast-like cells such as mouse osteoblast cells MC3T3-E1 migrate along a grating pattern, they undergo an 180° reversal of direction at apparently random moments [29]. More reversals appear to occur at discontinuities of the pattern, such as bends and ends, possibly due to the localized and temporary asymmetry between the cell leading and trailing edges induced by the abrupt changes of the microenvironment. Surfaces that harbour topographical patterns [52] or microimprinted adhesion molecules [53, 54] designed to introduce asymmetry to cell shape are known to influence cell speed and directionality.

In our previous study, we have observed cell directional reversals at the transitions of the angular grating pattern [29], but the influence of the bending angle and segment length is unknown. The precise design of the transitions that could promote cell migration directional reversals remains unexplored. In this study, we systematically examined how topographical features influence directional reversals of MC3T3-E1 cells, by using a series of V-shaped grating patterns. By varying the bending angles and segment lengths, we aimed to quantitatively assess the individual and combined effects of bends and ends on the migration behaviour, including speed, reversal rate, and cell morphology changes. We then explored the potential application of these effects in the design of substrates that function as switching gate to control the migration direction of cells.

## Materials and Methods

### Cell culture

MC3T3-E1 osteoblast cells were obtained from American Type Culture Collection (ATCC, CRL-2594) and were maintained in high glucose Dulbecco’s modified eagle medium (DMEM, Invitrogen), supplemented with 10% fetal bovine serum (FBS, Gibco), antibiotic antimycotic (Gibco; 100 units/m*l* penicillin G sodium, 100 mg/m*l* of streptomycin, and 0.25 mg/m*l* of Amphotericin B), and with 2 mM alanyl-L-glutamine (Gibco). The cells were incubated at 37°C in a 5% CO_2_ incubator and the medium was changed every 2 days.

### Platform design and fabrication

**Fig 1:**
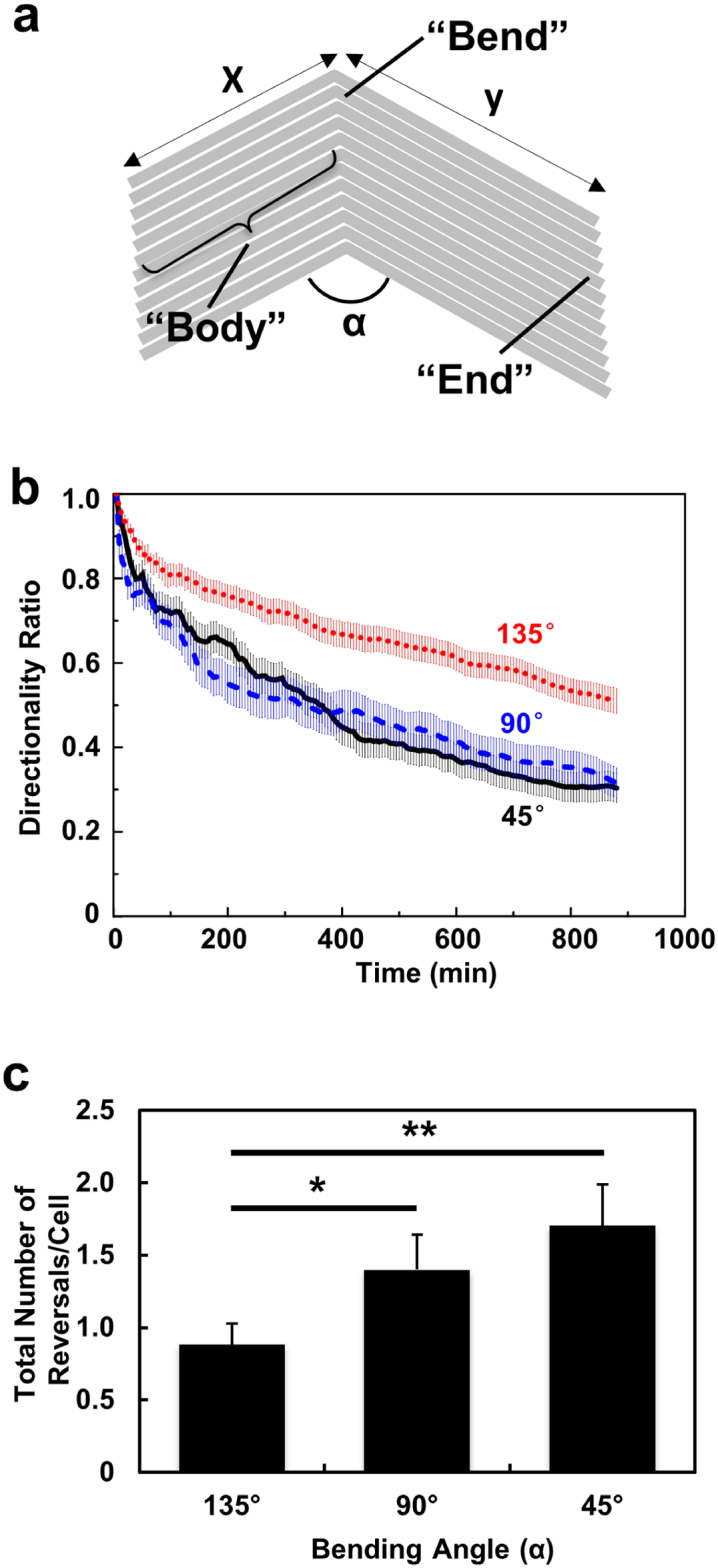
(**a**) Basic design of “V-shaped grating patterns” used in this study. (**b**) Directionality ratio of MC3T3-E1 cells on V-shaped grating patterns where bending angles are 45°, 90°, and 135°, respectively (N = 140, 3 independent experiments for 45° and 90° patterns, 4 independent experiments for 135° pattern). (**c**) Number of directional reversals per cell observed on these three patterns over 15 h (N = 140, one-way ANOVA with Dunnett’s post-hoc test, *p <0.05, **p <0.01).

Fig 1(a) summaries the design concept of the patterns used in this study, and the details are shown in S1 Fig. These patterns were fabricated by a molding technique as described before [29]. Briefly, a silicon (Si) mold was first fabricated using photolithography and dry etching. The Si mold was then coated with trichloro(1H, 1H, 2H, 2H-perfluorooctyl)silane (FOTS) which acted as an anti-sticking layer. The platform was fabricated with polydimethylsiloxane (PDMS), a biocompatible elastomer. The prepolymer was mixed with the curing agent (Dow Corning Sylgard 184 kit) with a mass ratio of 10:1. After degassing in a vacuum chamber, the mixture was poured on the Si mold and spin coated at 1000 rotation per minute (rpm). This left a substrate thickness of around 100 μm after baking for 2 h at 80°C on a hotplate. The PDMS platform was then peeled off from the Si mold, and treated with an O_2_ plasma for 3 min with a flow rate of 20 sccm O_2_, a chamber pressure of 80 mTorr, and an RF power of 55 W to make it hydrophilic. The surface energy was measured to be 71±3 mN/m using the two liquid (de-ionized water and cyclohexane) contact angle method.

### Time-lapse imaging

The patterned substrates were placed separately onto the 35 mm glass bottom confocal dishes (SPL), followed by 15 min sterilization with 95% ethanol. After washing thoroughly with phosphate buffered saline (PBS), MC3T3-E1 cells were seeded at a density of 7.5 × 10^3^ cells cm^-2^ onto the PDMS pattern. The cells were incubated on these patterns for approximately 5 h after seeding at 37°C and in 5% CO_2_ in a humidified incubator. Following the adherence of cells, the medium was replaced by the CO_2_-independent medium (Invitrogen 18045–088) supplemented with 10% FBS, antibiotic—antimycotic (100 U/m*l* of penicillin, 100 mg/m*l* of streptomycin, and 0.25 mg/m*l* of Amphotericin B), and 2 mM alanyl-L-glutamine. The cells were imaged on a laser scanning confocal microscope (Leica TCS SP5, with a 514 nm Ar visible laser) equipped with an incubation chamber at 37°C. Images were captured every 5 min over a period of 15 h. All results were analysed from at least 3 independent experiments in order to test the consistency of the results. The exact numbers of independent experiments for all the data were added in each figure caption.

### Immunofluorescence microscopy

After time lapse imaging of cell migration on the patterned substrates, MC3T3-E1 cells were washed thoroughly with PBS and fixed with 4% (w/v) freshly prepared paraformaldehyde (Sigma Aldrich) for 15 min at room temperature. Following fixation, the cells were quenched with 100 mM glycine in PBS for 15 min and washed twice with PBS (10 min each). Cells were then permeabilized in PBS containing 0.2% Triton X-100, and preblocked for 30 min with 1% bovine serum albumin in PBS at room temperature for 30 min. The cells were incubated with primary antibody mouse anti-vinculin (Millipore) for 2 h and washed twice in PBS. After washing, cells were incubated with secondary antibody conjugated to fluorochrome-conjugated Alexa 488 goat anti mouse (Invitrogen) for 2 h at room temperature. Then the cells were washed twice in PBS followed by counter staining with 0.165 M rhodamine-phalloidin (Invitrogen) and 1.09 M Hoechst 33342 (Sigma Aldrich) in PBS for 15 min. After washing twice in PBS, the cells were imaged on a confocal microscope (Leica TCS SPE).

### Data analysis

**Fig 2:**
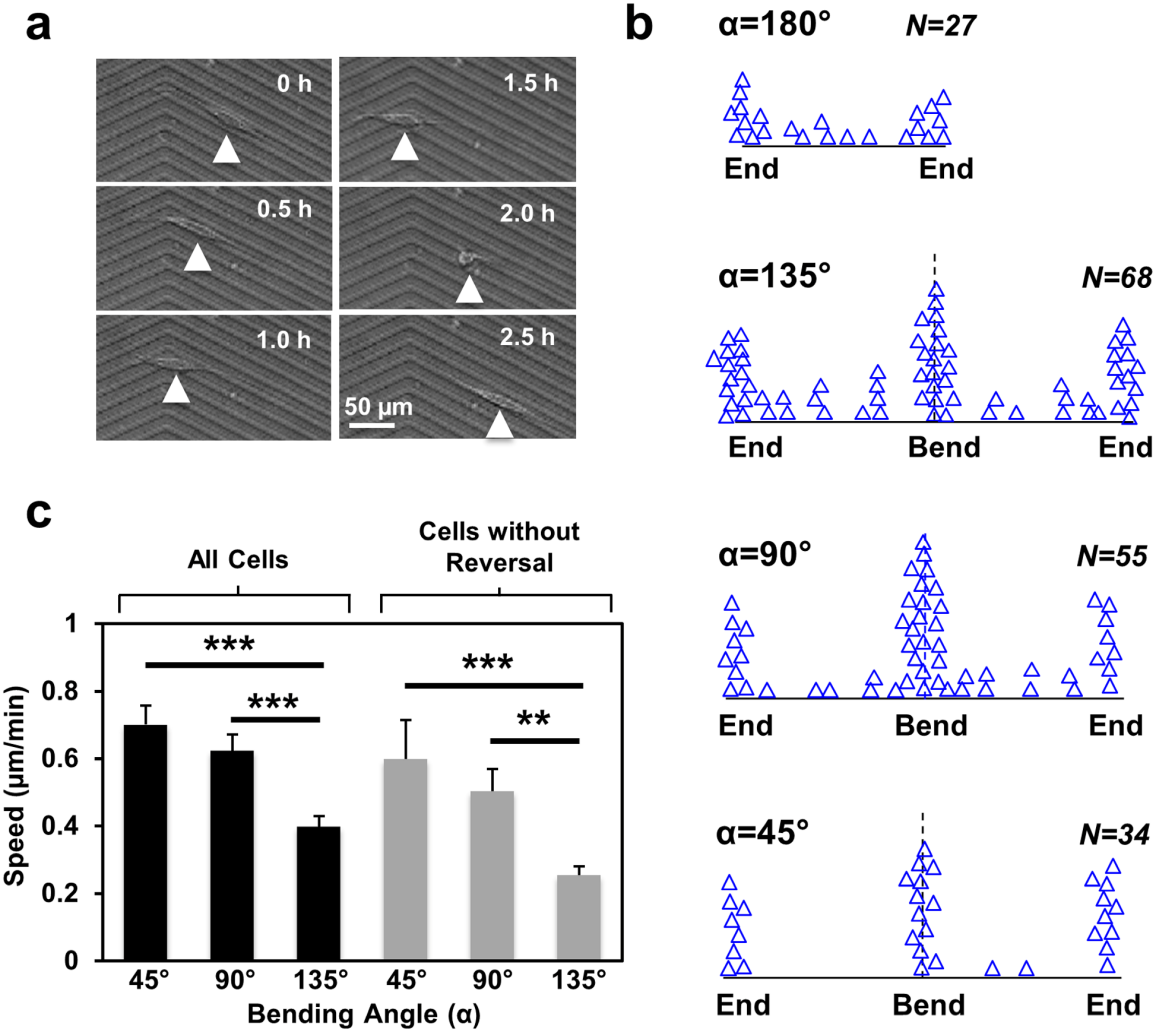
(**a**) Directional reversal of MC3T3-E1 cell at 135° bend. (**b**) Locations of directional reversals recorded on various V-shaped grating patterns. (**c**) Speed of cells with and without directional reversals on V-shaped grating patterns (N = 140, 3 independent experiments for 45° and 90° patterns, 4 independent experiments for 135° pattern, one-way ANOVA with Dunnett’s post-hoc test, **p <0.01, ***p <0.001).

**Fig 3:**
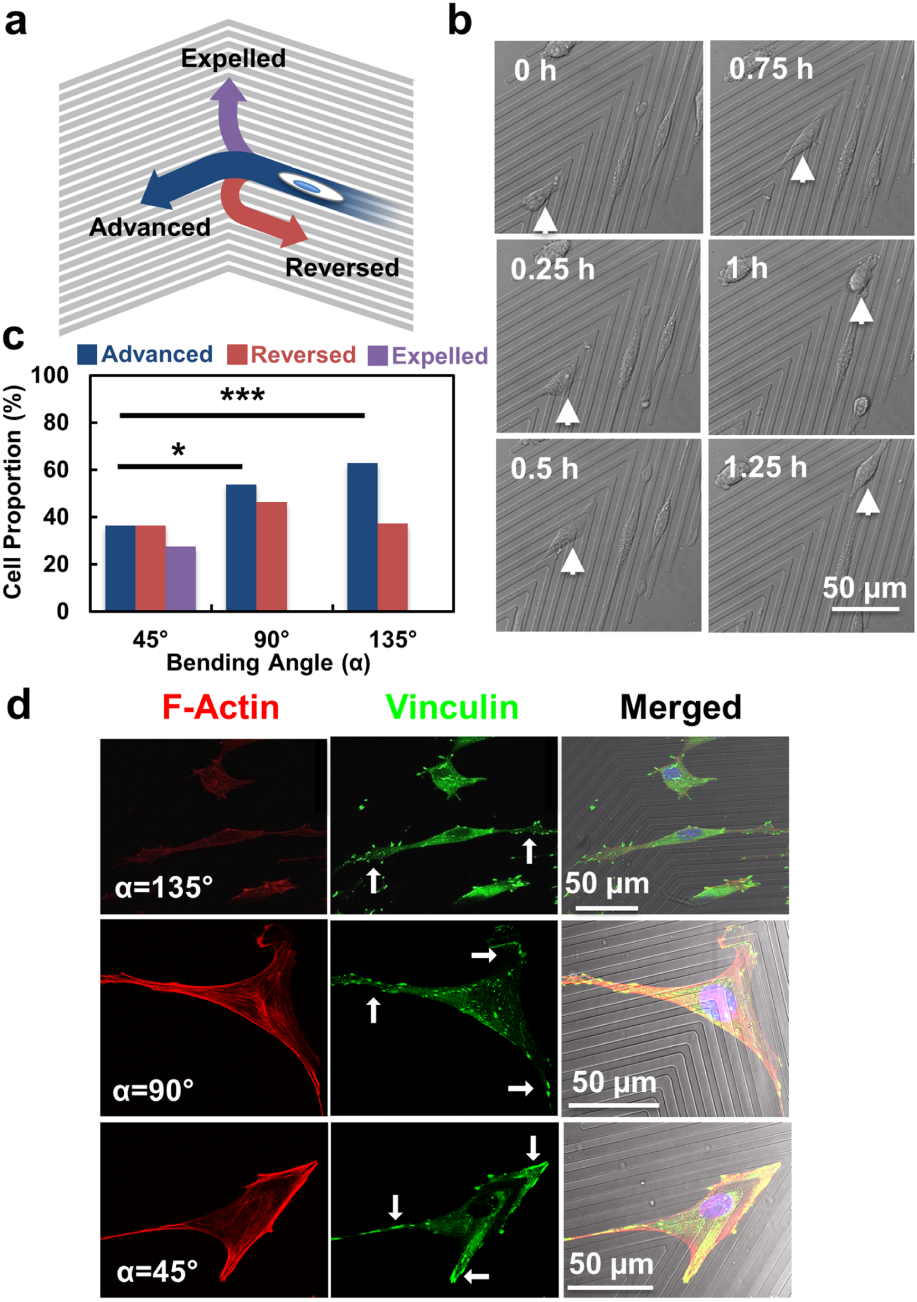
(**a**) Three possible routes taken by MC3T3-E1 cell approaching bend of V-shaped grating pattern. (**b**) “Expelled” cells at 45° bend. (**c**) Proportion of cells taking three possible routes on three V-shaped grating patterns (N = 140, 3 independent experiments for 45° and 90° patterns, 4 independent experiments for 135° pattern, chi-square test, *p <0.05, ***p <0.001). (**d**) Morphology of MC3T3-E1 cells at bends. Red: F-Actin, green: Vinculin.

**Fig 4:**
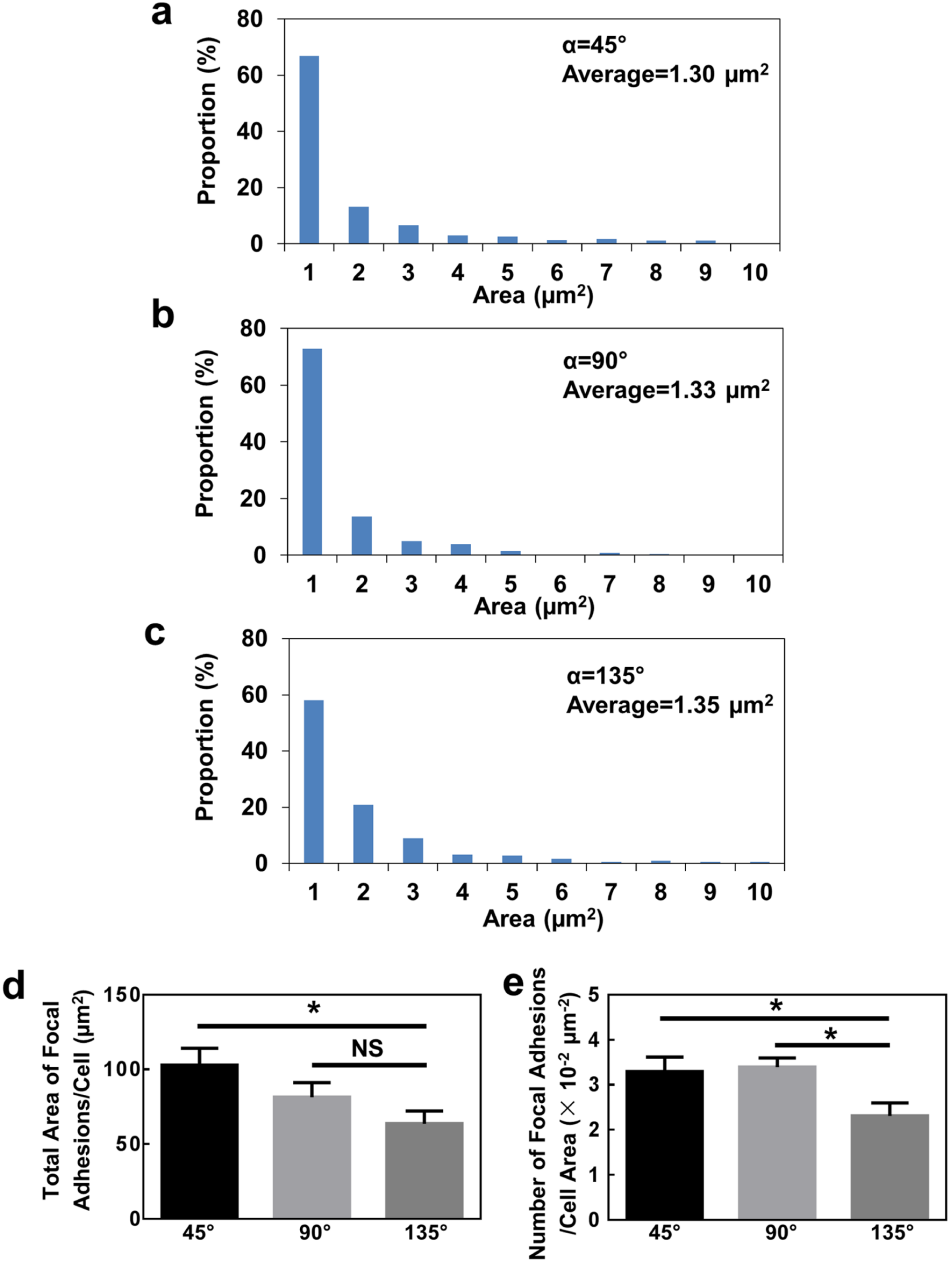
Histogram of focal adhesion sizes on V-shaped grating patterns with (**a**) 45°, (**b**) 90°, and (**c**) 135° bends. (**d**) Total focal adhesion area per cell and (**e**) number of focal adhesions per cell area of cells at bends (N = 15, one-way ANOVA with Dunnett’s post-hoc test, *p <0.05, NS denotes not significant).

**Fig 5:**
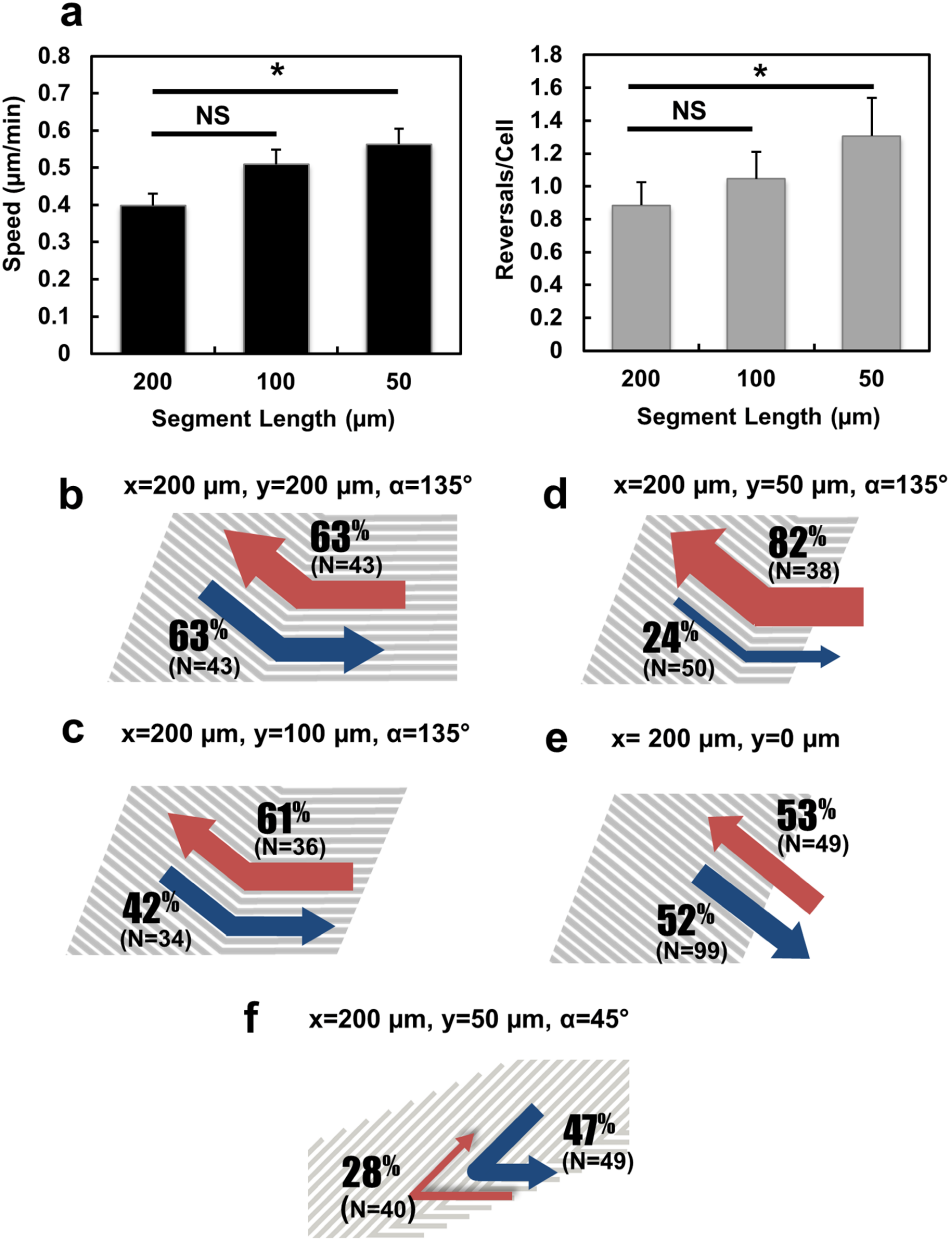
(**a**) Cell speed and number of reversal per cell on 135° V-shaped grating patterns with various segment lengths (N = 157, 5 independent experiments for 50 and 100 m patterns, 4 independent experiments for 200 m pattern, one-way ANOVA with Dunnett’s post-hoc test, *p <0.05, NS denotes not significant). Probability of cells that (**b**-**d**) advanced pass 135° bend of V-shaped patterns with various segment lengths from opposite directions, (**e**) moved in forward and reversed directions on straight gratings, and (**f**) advanced pass 45° bend of V-shaped pattern from opposite directions.

**Fig 6:**
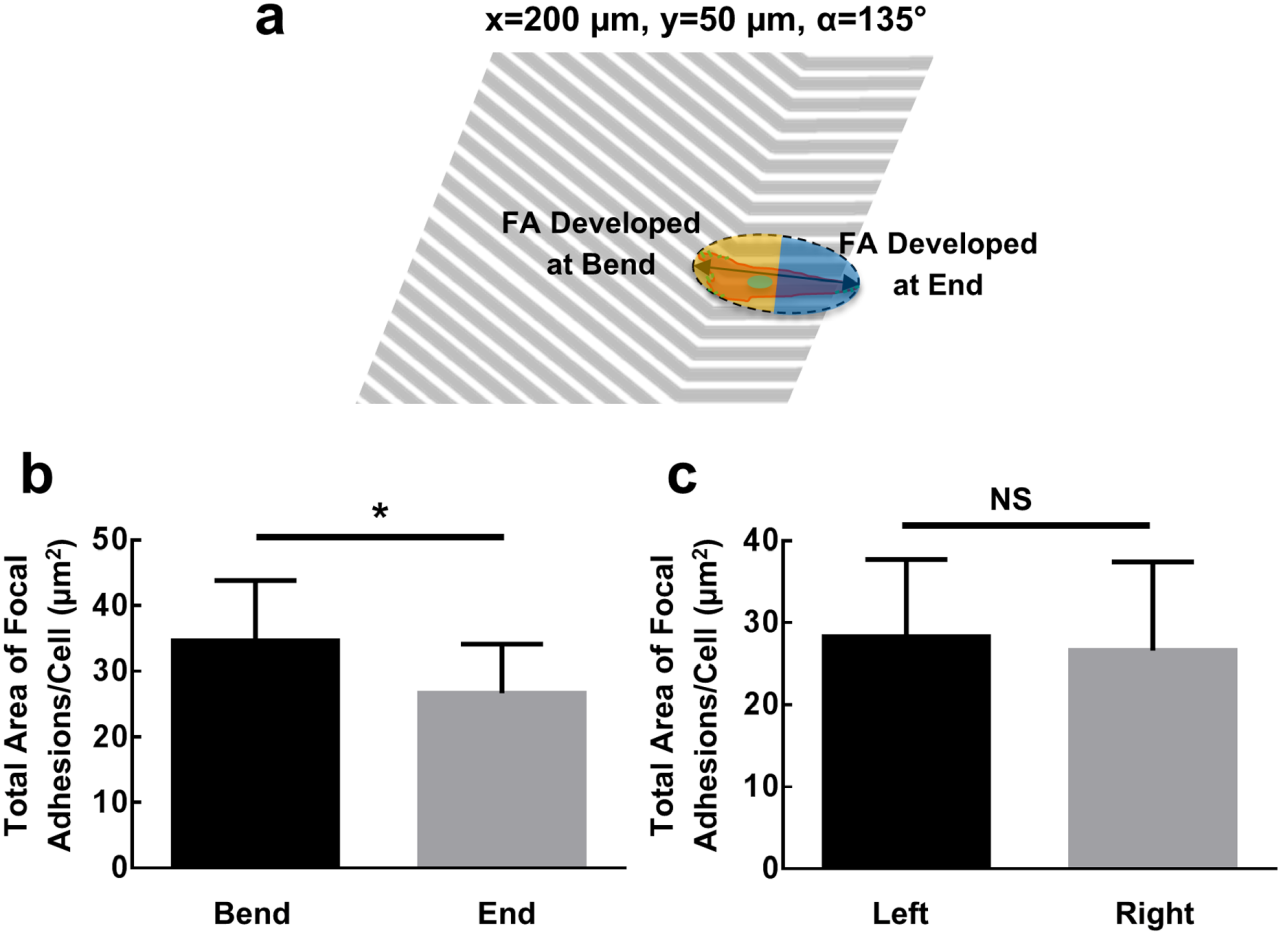
(**a**) Schematic diagram of cell touching both grating bend and end. (**b**) Total focal adhesion area developed at the side near grating bend and end (N = 14, ratio paired t-test, *p <0.05). (**c**) Total focal adhesion area developed at two ends of polarized cells on straight gratings (N = 16, ratio paired t-test, NS denotes not significant).

In this work, cells that did not divide or physically interact with other cells during the 15 h imaging period were selected for analysis. The migration tracks of these cells were obtained by using the Manual Tracking plugin of NIH ImageJ (version 1.48). The extracted position vectors were input to homemade Matlab codes where detailed analysis, including speed and directionality ratio, were calculated. All results are presented as mean ± standard error of the mean (sem). The directionality ratio is defined as:

directionality ratio=d/D (1)

where *d* is actual displacement of the cell (Euclidean distance between the initial and final positions) and *D* is the distance the cell travelled [55]. Both *d* and *D* are functions of time. Commercial software package GraphPad Prism (version 6.01) was used for performing statistical significance test where the null hypothesis was rejected at *p* <0.05. For the cell speed and number of reversals as shown in Figs 1(c) and 2(c), one-way analysis of variance (ANOVA) with Dunnett’s post-hoc analysis was performed. For Fig 3(c), chi-square test was used to compare the advanced cell proportion on patterns with 90° and 135° bends to the pattern with 45° bend. For the data shown in Figs 4(d), 4(e) and 5(a), ANOVA with Dunnett’s post-hoc analysis was adapted. For the total area of focal adhesions per cell as shown in Fig 6(b) and 6(c), ratio paired t-test was applied.

Total focal adhesion area per cell was assessed by ImageJ by summing up the area of all the focal adhesions [56]. Specifically, vinculin staining images were converted to 8-bit files, followed by image processing techniques for background removal and focal adhesion boundary enhancement, which were achieved by the built-in Subtract Background and Mask Unsharp functions. Images were then converted to binary format with a visually determined threshold to maximize the accuracy of focal adhesion sites. The resultant images were then processed by the built-in Analyze Particles function to obtain the area of each focal adhesion site.

## Results and Discussion

### V-shaped grating patterns prompted directional reversals of MC3T3-E1 cells

Fig 1(a) shows the basic design concept we used in this study. Grating patterns, in the form of parallel arrays of 5 μm wide and 1 μm tall ridges, were fabricated on a PDMS substrate as previously described [29]. Each ridge was varied from 250–400 μm in length and separated by 5 μm intervals. At certain location on this pattern, a single bend was introduced. In this study, different versions of this V-shaped grating pattern were used, with variations in two parameters: the sharpness of the bend (described by the angle α, Fig 1(a)) and the position of this bend relative to the ends (described by the segment lengths x and y, Fig 1(a)). The design and fabricated patterns used in this study can be found in S1 Fig. The dimensions of these patterns were designed so that most MC3T3-E1 cells would encounter the bend and at least one of the ends during a typical time-lapse imaging experiment (15 h). In this study, we focused our investigation on how geometrical features such as bends and ends influenced the migration of cells on the “body” of the pattern (Fig 1(a)), and therefore fixed the height and the pitch of the ridges to 1 and 5 μm, respectively. Fine adjustments of the system, including variations of these parameters as well as the physiochemical properties of the surface, will be a subject for future studies.

In consistent with previous observations, MC3T3-E1 cells generally migrated along the grating in the patterns, whereas cells on a flat PDMS surface moved in random-walk manner [52]. In the first series of experiments, we asked how the sharpness of the bend influenced the migration of MC3T3-E1 cells. We fabricated three patterns, in which the 400 μm long grating pattern was bent in the middle (x = y = 200 μm) at an angle (α) of 45°, 90°, or 135°, respectively (Patterns 1–3, S1 Fig). We tracked the trajectories of cells on these patterns (S2 Fig) and analysed the directional persistence of the cells by measuring the change of directionality ratio during the imaging period. The directionality ratio is defined as the Euclidean distance over total distance travelled, and this parameter has been used by other researchers to characterise migration directionality of both adherent and non-adherent cells with the presence of various kinds of ECM, e.g. topographic pattern and chemotactic gradient [55, 57–59]. This directionality ratio can be used to describe cell persistence, which includes many factors that give rise to directional changes. Cell directionality ratio decays more slowly on patterns with 135° bend than the 45° bend for geometrical reasons. This is due to the fact that when a cell passes an obtuse bend (135°), its direction is deviated by a smaller amount compared to an acute bend (45°). It is based on this geometrical effect that cell migration switch was designed, and the directionality ratio shows how effective the grating patterns could guide the cell migration direction (135° pattern more effective and more directional than the others). The time dependent directionality ratio for the 90° and 45° patterns showed little difference due to a proportion of cells (27.4% as shown in Fig 3(c)) were guided along the bending tips when they encountered the 45° bends, changing the direction by 22.5° only. Another factor that causes directional deviation is the cell reversals when they make a 180° turn. When a cell makes a directional reversal on the grating pattern, its Euclidean displacement decreases and leads to the reduced directionality ratio. If a cell migrates back to its original location, the directionality ratio becomes zero as the cell is not making a net displacement. Therefore, the directionality ratio is a useful indicator of how cell migration directionality could be controlled by the V-shaped gratings. We counted the number of directional reversals detected in the 15 h imaging period. As shown in Fig 1(c), significantly more directional reversals were recorded on the 45° and 90° patterns than on the 135° pattern (*p*<0.01 for 45° and *p*<0.05 for 90° bend). Hence, V-shaped grating patterns with more acute bends decreased migration persistence of cells by inducing more directional reversals.

### Acute bends influenced the overall migration properties of MC3T3-E1 cells

We noticed that many cells reversed their migration directions when they approached the bend and the ends (Fig 2(a)). This observation was confirmed when the locations of all directional reversals on these patterns over a 15 h period were mapped (Fig 2(b)). Predominately more reversals were detected around the bends and the ends than on the body of the patterns. Interestingly, the sharpness of the bend appeared to affect the migration behaviour of cells in regions away from the bend. Hence, in the absence of bend (α = 180°), even though directional reversals were preferentially occurred at the ends, some reversals were observed on the body. As the angle increased, however, reversals on the body were reduced. On the 45° pattern, reversals occurred almost entirely at the bends and the ends (Fig 2(b)).

We next examined the migration speed of MC3T3-E1 on the three patterns (Fig 2(c), black bars). Cells were migrating significantly faster on the patterns with 45° and 90° bends as compared to 135° bend (*p*<0.001). As MC3T3-E1 cells are known to temporarily speed up after directional reversals [29], the apparent increase in the average cell speed on patterns with sharper bends might be a reflection of the higher frequency of reversals on those patterns. To control this variability, we selectively analysed cells that did not exhibit any directional reversal during the imaging period (Fig 2(c), grey bars). As expected, these cells were slower than the reversing ones, regardless of the bending angle. Interestingly, even without experiencing any reversal, MC3T3-E1 cells still moved significantly faster on the patterns with more acute bends as compared to 135° bends (*p*<0.001 for 90° and *p*<0.01 for 45° bend). Hence, the V-shaped patterns not only induced local changes in the cells near the bend, but also led to long-range modifications such as the overall migration speed and directional reversals in positions away from the bend. As reported in the literature that focal adhesion size is highly related to cell motility [60], the higher cell speed on patterns with acute angles could be related to the larger number and area of the focal adhesions around the sharp tip of the bend.

### Morphological changes of cells caught at the bend

To investigate how the presence of an acute bend could exercise these long-range influences on cell migration, we examined the characteristics of cells at the bends (Fig 3). Our survey on an average of 140 cells on the three patterns indicated that cells adopted one of the following three routes when approaching the bend (Fig 3(a)): some cells were observed to reverse their directions at the bend (“Reversed”), and others migrated pass the bend to the other side of the pattern (“Advanced”). Some cells took a different route, migrating tangentially from the tip of the bend (“Expelled”), as illustrated in Fig 3(b). The “expelled” cells generally migrated along the tips over a distance of 100–200 m, before returning to the body on the either side of the bend. We measured the proportion of cells adopting these three routes at the bend of different sharpness (Fig 3(c)). Compared to patterns with 45° acute angle, cells tend to pass the bend as the angle becomes more obtuse, confirming that acute angle in the bend induces more directional deviations to the cells. Up to 37% of MC3T3-E1 cells reversed their directions when they reached a 135° bend and the rest of the cells advanced and passed the bend. At any given position on a straight grating with many possible reversal sites along the grating, directional reversals occurred at a very low frequency, confirming that the bend was a preferred site of reversals. As more cells reversed direction from the 90° bend (54%) than the 135° bend (37%), a sharper bend appeared to induce more directional reversals. However, at 45°, only 36% of the cells reversed. Not all the remaining cells advanced to the other side, though 27% of the cells were expelled from the tips of the bend (Fig 3(c)). As a comparison, no cell was observed to be expelled at the 90° bend. Fig 3(d) captures the morphology of MC3T3-E1 cells at the bends. At the 135° bend, cells retained a generally elongated morphology, with prominent filopodia established along the ridges on both sides of the bend. At 90° and 45° bends, however, cells often adopted a triangular shape, with focal adhesions detectable along both sides of the pattern and also at the tip of the bend. As cells tend to move along the directions parallel to the long axes of its most prominent focal adhesions [46], cells at the 45° bend demonstrated a similar chance of migrating to one of these three directions. Therefore, a sharper bend appears to cause more directional reversals, but if the bend becomes too acute, it induces a temporary deviation from the usual migration paths along the main axis of the grating.

The size of the focal adhesions located around the bends was quantitatively analysed. Fig 4(a)–4(c) show the histograms of the focal adhesion sizes on V-shaped grating patterns with bending angles varying from 45° to 135°. There was no significant difference in the average focal adhesion size for cells on grating patterns with these three bends. However, as shown in Fig 4(d) and 4(e), cells located around the 135° bends exhibited smaller total area of focal adhesions per cell and smaller number of focal adhesions per cell area compared to the other two patterns. These suggest that the designed bends induced morphological changes of cells and influenced the assembly of focal adhesions. As a result, cells on the 45° bending patterns, where cells developed larger total area and larger number of focal adhesions at the acute bends, migrated at higher speed as shown in Fig 2(c).

### The bend effect on cell migration was modified in proximity to the end

We interpret that the localized microenvironmental changes at the bend or the end may be the cause of the modifications of migration characteristics described above. We asked whether the effects of the bend and the end would be enhanced if they were brought closer together. To do this, we designed patterns (Patterns 3–5, S1 Fig) in which a single 135° bend was flanked by one segment of a constant length (x = 200 μm) and another segment of variable lengths (y = 50, 100, and 200 μm, respectively). As shown in Fig 5(a) (N = 157), the overall cell speed and the number of directional reversals observed over the 15 h imaging period increased as the length of segment decreased (i.e., as the 135° bend was closer to one end). This suggests that the combined effect of bends and ends was more pronounced when they were in close proximity. It should be pointed out that the “ends” refer to the locations where the grating pattern meets the flat surface. When a cell on the grating encounters the end, one part of the cell body will naturally extend onto the flat surface, while the rest of the cell still resides on the grating pattern. We then examined the behaviour of cells approaching the bend from the constant segment (x = 200 m). On the symmetrical V-shaped pattern (Fig 5(b), blue arrow), about 63% of cells advanced pass the 135° bend to the other segment (y = 200 m), with the remaining cells reversed direction from the bend. When the 135° bend was placed closer to the far end (y = 100 m), fewer cells could advance and pass it (42%, Fig 5(c), blue arrow). The percentage of “advanced” cells at the bend further decreased to 24% if the bend was even closer to the far end (y = 50 m, Fig 5(d), blue arrow). This suggests that reversal-inducing efficiency of a bend was also modified by the proximity of the end on the other side.

What about the cells that approached the bend from the ends with variable segment length? Since the 50 m segment was shorter than the length of many elongated MC3T3-E1 cells, we considered all the cells that approached the bend from the y segment, including cells that were previously from the flat surface outside the pattern. Fig 5(c) and 5(b) (red arrows) show that when the bend was 100 or 200 m away from the end, about 60% of cells could pass the bend and reach the other side of the pattern. However, when the bend was only 50 μm away from the end, this percentage was dramatically increased to 82% (Fig 5(d), red arrow). When the shorter segment was eliminated, thus turning the V-shaped pattern into a straight grating (Fig 5(e)), cells showed equal probability of moving in both forward and reversed directions. Although cells near the grating ends could sense different topography by their leading and trailing edges, they do not show preferences of migrating towards the grating pattern or escaping the grating pattern towards the flat surface when the segment length is at least 100 m long. Such a topographical discontinuity at the grating ends does not appear to influence cell migration direction unless the segment length is short where cells touch both the grating end and bend as shown in Fig 5(d).

### V-shaped gratings as unidirectional switching gates that control cell movement

The asymmetrical positioning of the 135° bend on the V-shaped grating pattern prevented the cells moving from the left to pass the bend (S3 Fig, left panels), but allowed the migration of cells from the right to pass through the bend (S3 Fig, right panels). This design of surface topography functions as a unidirectional switching gate. What happened when the angle of the overhang was changed? We designed a variation of this asymmetrical V-shaped pattern by changing the bending angle α to 45° (S1 Fig, Pattern 6). Only 28% of cells approaching the 45° bend from the short (50 m) segment side could advance to the long (200 m) segment side (Fig 5(f)). Therefore, bends situated at the same position (50 m from the short end) of the grating pattern can either pass (135°) or block (45°) cell migration, depending on the sharpness of the bending angle.

Although MC3T3-E1 fibroblast cells were used throughout this study, we have observed similar cell attachment, spreading, and movement from human osteosarcoma U2OS cells [29], as well as other cancer cells including non-metastatic lung cancer A549 cells, metastatic lung cancer H1299 cells, and HeLa cervical cancer cells (data not shown). It has also been reported that gratings, depending on the geometrical aspect ratio, tend to have a universal effect on cell alignment for different cells [61]. In general, the topographical bend induces cell direction switching applies to different cell types. However, the exact bending angle and segment length need to be optimized for a specific cell type for effective switching. On the other hand, a given bending topography may induce differential switching responses in different cells, allowing the bending structures to serve as cell-specific switching gates such as a cell sorter or filter without the need for fluorescent or magnetic particle labelling.

### Imbalanced distributions of focal adhesions in cells at the bends and ends

To investigate the mechanisms related to the cell switching gate, we examined the focal adhesions in cells located around the 50 μm overhangs of the grating structures with 135° bend. An ellipse was used to approximate the contour of a given cell located at both the bend and the end of the 50 m overhang as illustrated by the schematic diagram in Fig 6(a). The cell was divided into two halves based on the midpoint of the long axis of the approximating ellipse. Vinculin, the focal adhesion protein that anchors F-actin to the cell membrane and plays an important role in cell adhesion and migration, was stained and the total area of all the focal adhesions detected in each half was analysed as shown in Fig 6(b) [62]. Larger focal adhesion area was observed on the side of the cell near the bend compared to the side near the end. This is in accordance with the cell migration direction as a forward switch with the cell having a high probability of passing the bend and moving towards the longer segment as shown in Fig 5(d). For comparison, we also measured the total focal adhesion area of cells seeded on long straight gratings. No significant difference was observed between the total focal adhesion areas at the two sides of a given cell as shown in Fig 6(c). The exact mechanisms of how topographical bend and segment length, or pattern density, induce imbalanced focal adhesions at the leading and trailing ends of a cell are still under investigation.

Considering the full cell migration cycle including lamellipodia and filopodia protrusion or retraction, new adhesion sites formation, cytoplasm contraction, and membrane receptors recycling, it is believed that RhoA, Rac1, and Cdc42 GTPases, among others, play important roles for cell migration. They could control the signal pathways between cell surface receptors and related intracellular responses [7, 8, 63]. In our case, for grating structures with 135° bend and 50 m long segment at one end, 82% of the cells would advance pass the bend from the short to long segment, and 24% of the cells would advance pass the bend in the opposite direction. This unidirectional switching function could be due to the changes in cellular morphology when cells were migrating along the different positions of the grating structures. Such topographical cues were then picked up by the surface receptors, leading to re-distributions of cell focal adhesions as regulated by the series of GTPases proteins. For forward switching, when cells migrated to the 135° bend from the 50 m long segment, the size for the leading edge of the cells was larger compared to the trailing edge as the pattern density covered by the cells was higher around the bend than near the end of the 50 m long gratings. Likewise, the number and the total area of focal adhesions at the leading edge were larger than the trailing edge, which made the cells advanced pass the bend. When the cells were approaching from the opposite direction, the asymmetrical area between the leading and trailing edges of the cells resulted in cell reversals around the bend, and majority of the cells would not be able to advance beyond the bend.

Repelled cells, where cells moved along the tip of the bend, were only observed on grating structures with 45° bend. On top of the gratings with 45° bend, cells could also form large size cytoplasmic protrusions around the sharp bending tip, which became the third migration path along the tip. As shown in the immunofluorescence micrographs in Fig 3(d), cells at the bend formed a triangular morphology around the tip of 45° bend. It is believed that the changes in the area of the leading and trailing edges could lead to redistributions of the proteins. As a result, larger number and total size of focal adhesions were developed at the bending tip which made the cells expelled along the tip. The designs of the grating structure with different bending angles and segment lengths could be optimized to modify the cell shape with different degrees of symmetry between the leading and trailing edges. This will in turn change the distributions of various regulating proteins as the cells contact and move along the patterned surface. We are in the process of exploring the molecular basis of the directional reversal by transfecting cells with the corresponding plasmids to verify the localization and activity of the key regulators.

## Conclusions

In this study, we designed a single bend on a classical grating pattern and examined its effects on the migration of MC3T3-E1 cells. Our data reveal that the bend exerted a profound influence on the behaviour of cells, even in locations away from this topographical feature. The effect of the bend on cell migration appears to be defined by its sharpness and its proximity to the end. Our characterisation of these two factors allowed us to design simple geometric shapes that exercised powerful control over the migration of adherent cells in culture. A pattern with an asymmetrically situated 135° bend will potentially allow the movement of cells from one side of the substrate to the other side, but block the movement of cells in an opposite direction. Hence this relatively simple pattern can be used as a unidirectional switching gate for cell movement. Remarkably, by increasing the sharpness of the bend to 45°, the directionality of control is reversed.

Most of the existing devices for one-way movement of cells achieve this by controlling flow dynamics in microfluidic channels [64–66]. The use of liquid flow in these systems prevents the application on adherent cells and cells that are functional influenced by the sheer forces of the surrounding environment [67, 68]. In our system, cells were cultured on an open substrate and without any manipulation of medium flow. Instead, the redirection of cells on the substrate was driven by the forces produced internally by the cells, which are dictated by the asymmetry of focal adhesive sites caused by the topographical structures of the surface. Since our system utilises the endogenous adhesion and cytoskeletal mechanisms of the cell, we hypothesise that cell types with distinct sizes and migration properties will react differently to these patterns, hence pathing the way for a cell type-specific unidirectional filter. We envisage that the data presented in this study will be the basis of more complex “smart” platform, which could function as a self-organising system that can “sort” adherent cells to predesigned locations of the substrate.

## Supporting Information

S1 Fig Pattern 1–3: Symmetrical angular gratings with 45°, 90°, and 135° bending angles, and 200 m long segments on both sides. Pattern 4–5: Asymmetrical angular gratings with 135° bending angle, 200 m long segment on left, 100 m and 50 m long segment on right. Pattern 6: Asymmetrical angular gratings with 45° bending angle, 300 m long segment on left and 50 m long segment on right.
(TIF)

S2 Fig Tracking of cell migration on 135° symmetrical angular gratings. (TIF)

S3 Fig Differential migration characteristics of MC3T3-E1 cells approaching same V-shaped pattern (α = 135°, x = 200 m, y = 50 m) from opposite directions. (TIF)
