# Supplementary figures and images for: A Unidirectional Cell Switching Gate by Engineering Grating Length and Bending Angle
Source: PLoS One. 2016 Jan 28;11(1):e0147801. doi: 10.1371/journal.pone.0147801 (PMC4731054; doi:10.1371/journal.pone.0147801)

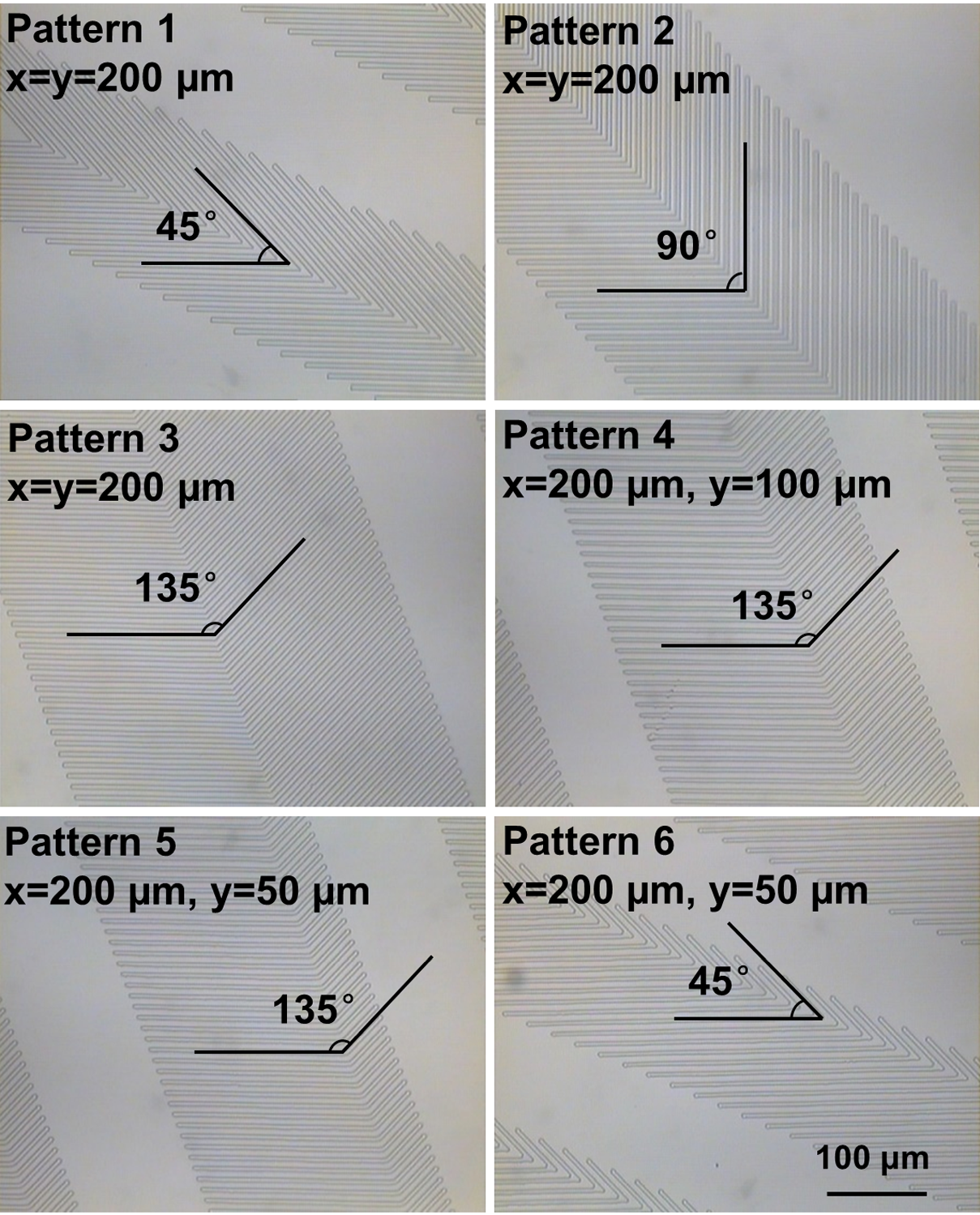

Supplement: S1 Fig — Pattern 1–3: Symmetrical angular gratings with 45°, 90°, and 135° bending angles, and 200 m long segments on both sides. Pattern 4–5: Asymmetrical angular gratings with 135° bending angle, 200 m long segment on left, 100 m and 50 m long segment on right. Pattern 6: Asymmetrical angular gratings with 45° bending angle, 300 m long segment on left and 50 m long segment on right. (TIF) [file pone.0147801.s001.tif]

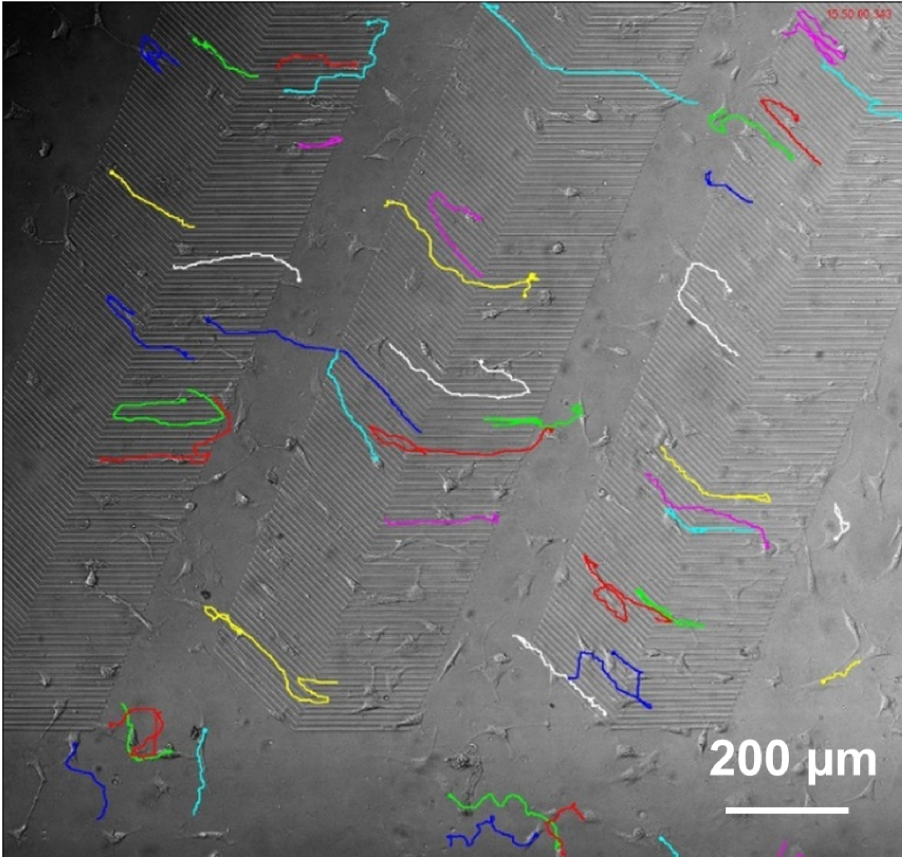

Supplement: S2 Fig — (TIF) [file pone.0147801.s002.tif]

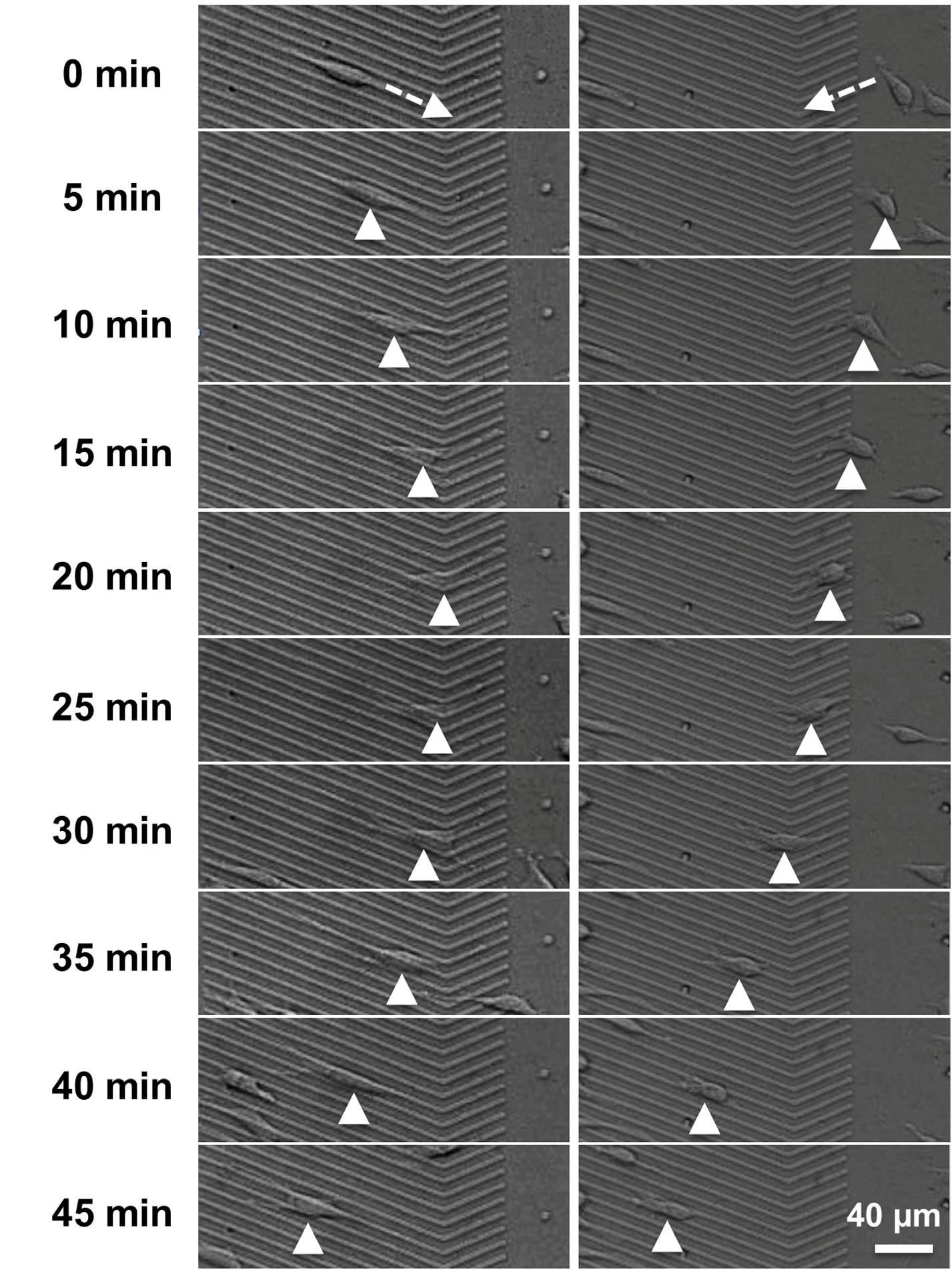

Supplement: S3 Fig — (TIF) [file pone.0147801.s003.tif]
